# Dietary habits, traveling and the living situation potentially influence the susceptibility to SARS-CoV-2 infection: results from healthcare workers participating in the RisCoin Study

**DOI:** 10.1007/s15010-024-02201-4

**Published:** 2024-03-04

**Authors:** Paul R. Wratil, Thu Giang Le Thi, Andreas Osterman, Irina Badell, Melanie Huber, Ana Zhelyazkova, Sven P. Wichert, Anna Litwin, Stefan Hörmansdorfer, Frances Strobl, Veit Grote, Tarek Jebrini, Helga P. Török, Veit Hornung, Alexander Choukér, Berthold Koletzko, Kristina Adorjan, Sibylle Koletzko, Oliver T. Keppler, the RisCoin study group

**Affiliations:** 1 Max von Pettenkofer Institute and Gene Center, Virology, National Reference Center for Retroviruses, LMU Munich, Pettenkoferstr. 9a, 80336 Munich, Germany; 2 German Center for Infection Research (DZIF), Partner Site Munich, Munich, Germany; 3 Department of Pediatrics, Dr. von Hauner Children’s Hospital, LMU University Hospital, LMU Munich, Lindwurmstraße 4, 80337 Munich, Germany; 4 Institut für Notfallmedizin und Medizinmanagement (INM), LMU University Hospital, LMU Munich, Munich, Germany; 5 Department of Psychiatry and Psychotherapy, LMU University Hospital, LMU Munich, Nussbaumstraße 7, 80336 Munich, Germany; 6 Bavarian Health and Food Safety Authority, Oberschleissheim, Germany; 7 Department of Neurology, LMU University Hospital, LMU Munich, Munich, Germany; 8 Gene Center and Department of Biochemistry, LMU Munich, Munich, Germany; 9 Department of Anesthesiology, Laboratory of Translational Research Stress and Immunity, LMU University Hospital, LMU Munich, Munich, Germany; 10 Institute of Psychiatric Phenomics and Genomics (IPPG), LMU University Hospital, LMU Munich, Munich, Germany; 11 Center for International Health (CIH), LMU Munich, Munich, Germany; 12 University Hospital of Psychiatry and Psychotherapy, University of Bern, Bern, Switzerland; 13 Department of Pediatrics, Gastroenterology and Nutrition, School of Medicine Collegium Medicum, University of Warmia and Mazury, Olsztyn, Poland

**Keywords:** SARS-CoV-2, COVID-19, Healthcare workers, General population, Risk, Prevention

## Abstract

**Purpose:**

To explore occupational and non-occupational risk and protective factors for the coronavirus disease 2019 (COVID-19) in healthcare workers (HCWs).

**Methods:**

Serum specimens and questionnaire data were obtained between October 7 and December 16, 2021 from COVID-19-vaccinated HCWs at a quaternary care hospital in Munich, Germany, and were analyzed in the RisCoin Study.

**Results:**

Of 3,696 participants evaluated, 6.6% have had COVID-19 at least once. Multivariate logistic regression analysis identified working in patient care occupations (7.3% had COVID-19, 95% CI 6.4–8.3, *P*_r_ = 0.0002), especially as nurses, to be a potential occupation-related COVID-19 risk factor. Non-occupational factors significantly associated with high rates of the disease were contacts to COVID-19 cases in the community (12.8% had COVID-19, 95% CI 10.3–15.8, *P*_r_ < 0.0001), being obese (9.9% had COVID-19, 95% CI 7.1–13.5, *P*_r_ = 0.0014), and frequent traveling abroad (9.4% had COVID-19, 95% CI 7.1–12.3, *P*_r_ = 0.0088). On the contrary, receiving the basic COVID-19 immunization early during the pandemic (5.9% had COVID-19, 95% CI 5.1–6.8, *P*_r_ < 0.0001), regular smoking (3.6% had COVID-19, 95% CI 2.1–6.0, *P*_r_ = 0.0088), living with the elderly (3.0% had COVID-19, 95% CI 1.0–8.0, *P*_r_ = 0.0475), and frequent consumption of ready-to-eat meals (2.6% had COVID-19, 95% CI 1.1–5.4, *P*_r_ = 0.0045) were non-occupational factors potentially protecting study participants against COVID-19.

**Conclusion:**

The newly discovered associations between the living situation, traveling as well as dietary habits and altered COVID-19 risk can potentially help refine containment measures and, furthermore, contribute to new mechanistic insights that may aid the protection of risk groups and vulnerable individuals.

**Supplementary Information:**

The online version contains supplementary material available at 10.1007/s15010-024-02201-4.

## Introduction

Since its emergence, the coronavirus disease 2019 (COVID-19) caused by the severe acute respiratory syndrome coronavirus 2 (SARS-CoV-2) has been a substantial burden to global health and, as of January 2024, led to approximately 7 million deaths as well as countless cases of severe disease [1]. Despite the majority of the population either being vaccinated against COVID-19 or having experienced a SARS-CoV-2 infection at least once [2–4], infection, re-infection and breakthrough infection (BI) still pose a considerable risk for severe and fatal illness [5, 6], especially among immunocompromised individuals [7, 8]. Furthermore, due to the rapid evolution of SARS-CoV-2, it is conceivable that new virus variants will surface in future that display enhanced immune escape and increased virulence leading to an elevated disease burden and rising numbers of severely affected individuals as well as COVID-19-associated deaths [9].

Therefore, in-depth analysis of risk and protective factors for SARS-CoV-2 infection, re-infection and BI remains important to identify vulnerable individuals and refine the application of containment measures. Healthcare workers (HCWs) are not only at the forefront of treating SARS-CoV-2-infected individuals, but also are essential to all patient care. Protecting this occupational group from COVID-19, thus, has been and is key to prevent severe distress to the healthcare system. However, adequate protection of HCWs requires the thorough investigation of occupational risk and protective factors for COVID-19. Simultaneously, healthcare professionals are a large and diverse occupational group, among others, including various age groups, education levels, health conditions, and lifestyles. Consequently, non-occupational factors modulating the COVID-19 risk in HCWs are likely also applicable for the general population and insight gained may potentially trigger investigations into novel mechanisms underlying antiviral immunity.

To research COVID-19 risk and protective factors in HCWs, we recruited a cohort of at least double-vaccinated HCWs at a quaternary care hospital in Munich, Germany, to donate samples for serological analysis and answer a questionnaire including epidemiological, COVID-19- and lifestyle-specific items as part of the RisCoin Study [10]. Using this cross-sectional approach, we identified several occupational and non-occupational factors that modulated the risk of participants for SARS-CoV-2 infection, re-infection, and BI, some of which have not been reported to date.

## Materials and methods

### Study design, setting and participants

**Fig. 1:**
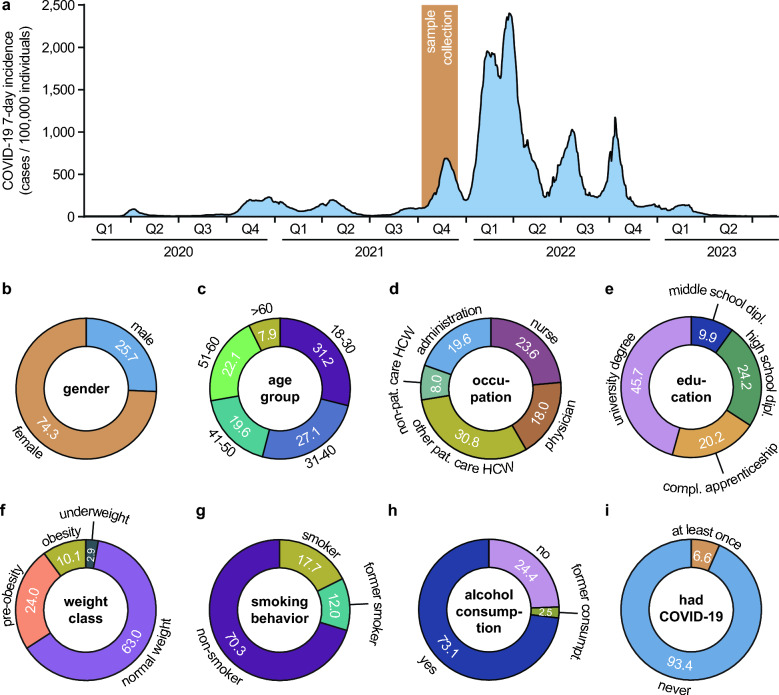
Overview of the study period and study population. **A** 7-day incidence of COVID-19 as cases per 100,000 individuals in Bavaria between March 1, 2020, and August 17, 2023 (blue) [39]. Serum samples from participants were collected between October 7 and December 16, 2021 (orange). **B**–**I** Information on the study population shown as percentages of all participants: **B** gender, **C** age group, **D** occupation within the hospital, **E** formal education, **F** weight class calculated from the participants’ body mass index according to the definitions for adults by the World Health Organization [40], **G** smoking behavior, **H** alcohol consumption, **I** had COVID-19, defined as being positive for nucleocapsid-specific anti-SARS-CoV-2 antibodies and/or reporting of ever having been tested positive for an acute SARS-CoV-2 infection by PCR. *compl.* completed, *dipl.* diploma, *pat.* patient

The recruiting process and the basic characterization of the RisCoin Study were described recently [10]. Briefly, between October 7 and December 16, 2021, during the fourth wave of the COVID-19 pandemic in Germany (Fig. 1A), we invited all employees at the University Hospital in Munich, (LMU Klinikum) the second largest quaternary care hospital in Germany, to donate a serum sample and answer an online questionnaire. Most HCWs were enrolled into the study during 3 weeks in October and 2 weeks in December 2021, when the recruitment was combined with a COVID-19 vaccination campaign organized by the hospital. All participants had received at least two COVID-19 vaccinations prior to enrollment.

### Data collection

Serum samples were tested for SARS-CoV-2-specific anti-nucleocapsid antibodies as an indicator for either sub-acute or resolved COVID-19. The online questionnaire comprised various items assessing, among others, demographics, occupational situation, perceived exposure to SARS-CoV-2, COVID-19 vaccination status, health conditions, dietary habits, and lifestyle (Supplementary Table 1).

### Nucleocapsid-specific anti-SARS-CoV-2 antibody detection assay

Blood samples were centrifuged for 10 min at 3200 × g and room temperature. Subsequently, at least 500 µL serum were extracted from each specimen. The Elecsys Anti-SARS-CoV-2 assay (Roche, Basel, Switzerland, cat.: 09203095190) was performed according to the manufacturer’s instructions to determine whether the serum samples were positive for nucleocapsid-specific anti-SARS-CoV-2 antibodies [11].

### Data analysis

Participants were defined as having had COVID-19 at least once if they were positive for nucleocapsid-specific anti-SARS-CoV-2 antibodies and/or reported ever having been tested positive for an acute SARS-CoV-2 infection by PCR. Individuals who reported being vaccinated with a vaccine that induces nucleocapsid-specific anti-SARS-CoV-2 antibodies (e.g., CoronaVac COVID-19 vaccine, Sinovac Biotech, Beijing, China) were excluded from data evaluation.

Data were analyzed using SAS Enterprise Guide 8.1 and SAS 9.4 (Statistical Analysis Software, SAS Institute Inc., Cary, NC, USA). Binominal 95% confidence intervals (95% CI) were calculated using the Wilson score interval. Differences between categorical variables were tested for their statistical significance using Fisher’s exact test, in case of multiple comparisons, with Holm’s multiple testing correction. Multivariate analysis was performed using a logistic regression adjusted for gender and age followed by recursive feature elimination up to a threshold of *P* = 0.05 with the status of having had COVID-19 upon study inclusion as the dependent variable and the answers from different study questionnaire items as independent variables. 95% Wald confidence intervals were calculated for odds ratios in multivariate regression analysis.

## Results

### Characteristics of the study population

Participants were recruited between October 7 and December 16, 2021, which was coinciding with the fourth wave of the COVID-19 pandemic in Bavaria (the study site) dominated by SARS-CoV-2 variant of concern (VoC) delta (Fig. 1A). Of the 3,816 HCWs included in the RisCoin cohort, 3,696 individuals met the criteria for further data evaluation. The relative distributions of gender, age, occupation, formal education, body mass index, and alcohol consumption in the study cohort are depicted in Fig. 1B–H.

Defined by being positive for nucleocapsid-specific anti-SARS-CoV-2 antibodies and/or reporting of ever having been tested positive for an acute SARS-CoV-2 infection by PCR, 6.6% (245/3,687) of the study cohort have had COVID-19 at least once upon inclusion into the study (Fig. 1I). Thereof, 50.2% (123/245) had detectable anti-nucleocapsid antibodies and reported a positive SARS-CoV-2 PCR test in the past, 31.4% (77/245) had anti-nucleocapsid antibodies but were never tested PCR-positive, and 18.4% (45/245) were seronegative but indicated at least one positive PCR test result.

At the time of blood sampling, 7.6% (282/3,696) of the cohort had received a third COVID-19 vaccination, whereas 92.4% (3,414/3,696) were vaccinated twice. For their basic immunization, 94.2% (3,482/3,696) of participants were vaccinated with two consecutive doses of the mRNA vaccine BNT162b2 (Pfizer-BioNTech, New York, USA).

### COVID-19 risk by gender, age, and medical conditions

**Fig. 2:**
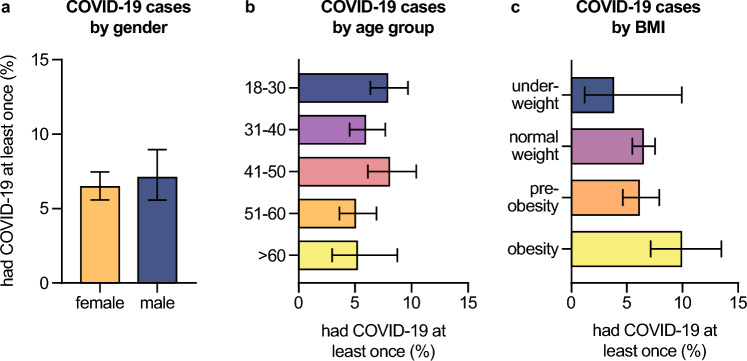
COVID-19 risk by gender, age, and weight class. Percentages of all participants who had COVID-19 at least once (defined as being positive for nucleocapsid-specific anti-SARS-CoV-2 antibodies and/or reporting of ever having been tested positive for an acute SARS-CoV-2 infection by PCR) by **A** gender, **B** age group, **C** weight class calculated from the participants’ body mass index. Error bars indicate 95% confidence intervals obtained from Wilson corrected binominal testing. *P* values next to brackets indicate statistically significant differences between groups calculated using Fisher’s exact test (**A**) or Fisher’s exact test with Holm’s multiple testing correction (**B**, **C**). If no *P* values are given, no statistically significant differences were detected

There was no significant difference comparing the COVID-19 rates of female (6.5%, 95% CI 5.6–7.5) and male participants (7.1%, 95% CI 5.6–9.0), (Fig. 2A). Similarly, age had no influence on the COVID-19 risk, albeit individuals ranging from 18 to 30 and 41 to 50 years of age had, by tendency, higher rates of sub-acute or resolved COVID-19 (7.9%, 95% CI 6.4–9.7, and 8.0%, 95% CI 6.1–10.4, respectively) compared to other age groups (Fig. 2B). There was a trend towards elevated COVID-19 risk with increasing body mass index: 3.8% (95% CI 1.2–9.9) of underweight participants have had COVID-19, 6.4% (95% CI 5.5–7.5) of those with normal body weight, 6.1% (95% CI 4.6–7.9) of pre-obese, and 9.9% (95% CI 7.1–13.5) of obese individuals (Fig. 2C). In line with this result, we found that obesity was significantly associated with increased COVID-19 rates in our multivariate data analysis (*P*_r_ = 0.0014, Table 1).

**Table 1:** Potential COVID-19 risk factors and protective factors among study participants that are significant in multivariate analysis

		Had COVID-19 (%)	(95% CI)	Had COVID-19/total	*P*_r_-value
	All participants	6.6	(5.9–7.5)	245/3,696	
Risk factors	Community contact to infected individual	12.8	(10.3–15.8)	77/600	< 0.0001
	Stronger adverse effects after 1st vaccination	11.2	(5.8–20.1)	10/89	0.0255
	Obesity	9.9	(7.1–13.5)	37/374	0.0014
	≥ 4x/year traveling to foreign country	9.4	(7.1–12.3)	50/533	0.0088
	Patient care HCW	7.3	(6.4–8.3)	194/2,665	0.0002
Protective factors	Early basic immunization (before June 1, 2021)	5.9	(5.1–6.8)	168/2,838	< 0.0001
	Daily smoking	3.6	(2.1–6.0)	15/416	0.0088
	Living in household with elderly (> 60 years)	3.0	(1.0–8.0)	4/133	0.0475
	Frequent consumption of ready-to-eat meals	2.6	(1.1–5.4)	7/273	0.0045

Binominal 95% confidence intervals (95% CI) were calculated using the Wilson score interval. Logistic regression adjusted to gender and age followed by recursive feature elimination up to a threshold of *P* = 0.05. *HCW* healthcare worker, *P*_*r*_*-value* *P *value for multivariate logistic regression analysis

Next, we investigated whether certain self-reported medical conditions influenced the COVID-19 risk. Neither cardiovascular, pulmonary, renal, rheumatological, hepatic and gastrointestinal diseases, nor diabetes mellitus, metabolic as well as neurological disorders, thyroid dysfunction, cancer, and allergies had a statistically significant effect on the fraction of individuals who have had COVID-19 (Supplementary Table 1). Immunosuppressive therapy at the time of inclusion also had no significant influence on COVID-19 rates (Supplementary Table 1).

### Occupational and vaccination-dependent COVID-19 risk and protective factors

**Fig. 3:**
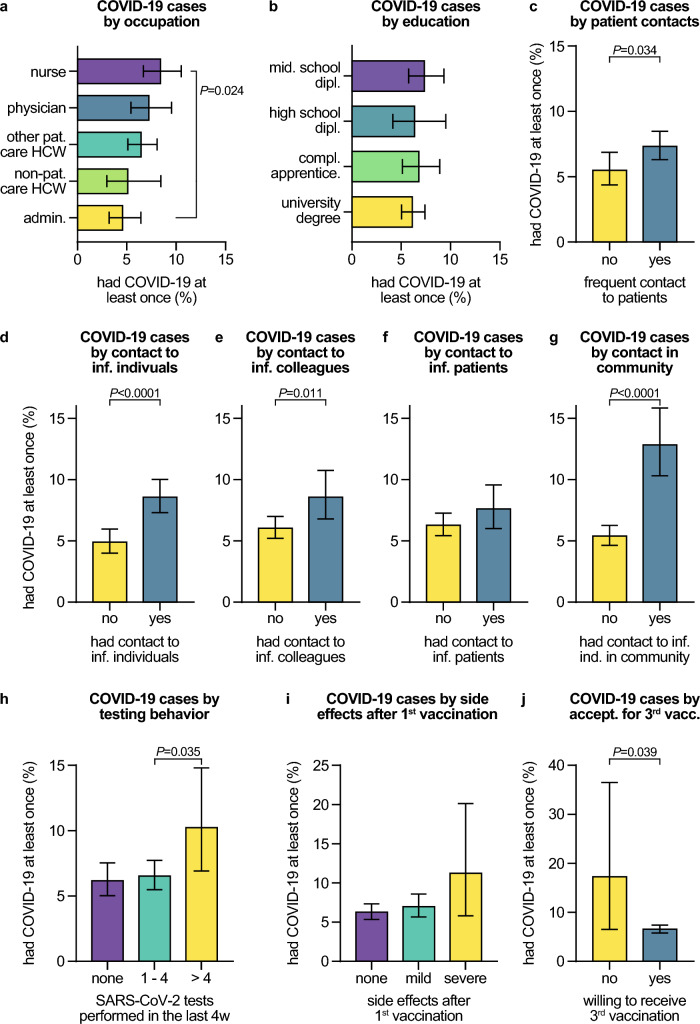
Occupational and vaccination-dependent COVID-19 risk factors. Percentages of all participants who had COVID-19 at least once (defined as being positive for nucleocapsid-specific anti-SARS-CoV-2 antibodies and/or reporting of ever having been tested positive for an acute SARS-CoV-2 infection by PCR) by **A** occupation within the hospital, **B** formal education, **C** reporting of regular face-to-face contacts to patients, to SARS-CoV-2-infected individuals **D** in general, **E** among colleagues, **F** among patients in the hospital or **G** outside the hospital in the community, **H** by testing behavior in the previous four weeks, **I** vaccination status, **J** acceptance for the 3rd COVID-19 vaccination among double-vaccinated participants. Error bars indicate 95% confidence intervals obtained from Wilson corrected binominal testing. *P* values next to brackets indicate statistically significant differences between groups calculated using Fisher’s exact test (**C**–**G**, **I**, **J**) or Fisher’s exact test with Holm’s multiple testing correction (**A**, **B**, **H**). If no *P* values are given, no statistically significant differences were detected. *admin.* administration, *apprentice.* apprenticeship, *dipl.* diploma, *ind.* individuals, *inf.* infected, *mid.* middle, *pat.* patient, *vacc.* vaccination

Investigating different occupations within the hospital, we noted significantly higher COVID-19 rates among nurses (8.4%, 95% CI 6.7–10.5) compared to HCWs working in administration (4.6%, 95% CI 3.2–6.4, *P* = 0.014, Fig. 3A). Differences in the fractions of individuals with previous COVID-19 between occupational groups were unlikely due to varying educational backgrounds (Fig. 3B). However, HCWs with regular contact to patients had significantly higher COVID-19 rates compared to those without patient contact. (7.3%, 95% CI 6.3–8.5, vs. 5.5%, 95% CI 4.4–6.9, respectively, *P* = 0.034, Fig. 3C). Supporting this, our multivariate analysis showed that HCWs in patient care occupations were at increased COVID-19 risk (*P*_r_ = 0.0002, Table 1).

Participants who reported ever having experienced a close contact to a SARS-CoV-2-infected individual have had COVID-19 more frequently compared to others (8.6%, 95% CI 7.3–10.0, vs. 4.9%, 95% CI 4.0–6.0, respectively, *P* < 0.0001, Fig. 3D). Investigating these close contacts in more detail, we uncovered that HCWs with exposure to SARS-CoV-2-infected colleagues at work had higher rates of COVID-19 (8.6%, 95% CI 6.8–10.7) compared to those who did not (6.0%, 95% CI 5.2–7.0, *P* = 0.011, Fig. 3E). Occupation-related exposure to COVID-19 patients, however, did not increase the COVID-19 risk in participants (Fig. 3F). Particularly high rates of HCWs who have had COVID-19 were observed among employees who reported experiencing close contacts to SARS-CoV-2-infected individuals outside of the hospital, in the community, (12.8%, 95% CI 10.3–15.8) compared to those who indicated no such community contacts (5.4%, 95% CI 4.6–6.3, *P* < 0.0001, Fig. 3G). Corroborating this result, we found that exposure to SARS-CoV-2 in the community was a statistically significant COVID-19 risk factor in our multivariate data analysis (*P*_r_ < 0.0001, Table 1).

We examined whether the frequency of getting tested within the previous month before inclusion influenced the COVID-19 risk in the study cohort. Intriguingly, participants who got PCR-tested more than four times within the last 4 weeks had higher COVID-19 rates (10.2%, 95% CI 6.9–14.8) compared to those who got tested less often (6.5%, 95% CI 5.5–7.3, *P* = 0.035, Fig. 3H).

We observed a trend towards higher COVID-19 rates in HCWs who indicated experiencing more intense adverse effects after the first vaccination resulting in absence from work for at least one day (11.2%, 95% CI 5.8–20.1) than in HCWs with no (6.3%, 95% CI 5.3–7.3) or milder adverse effects (7.0%, 95% CI 5.7–8.6, Fig. 3I). In line with this result, we discovered that such more severe adverse effects after the first vaccination were associated with an increased rate of sub-acute or resolved COVID-19 in multivariate analysis (*P*_r_ = 0.0255, Table 1). No such trend, however, was found after the second and third vaccinations (Supplementary Table 1). Twice-vaccinated participants who were reluctant towards receiving a third COVID-19 vaccination had significantly higher COVID-19 rates (17.2%, 95% CI 6.5–36.5) compared to those who were willing to receive the third shot (6.5%, 95% CI 5.7–7.6, *P* = 0.039, Fig. 3J). On a different note, our data analysis showed that HCWs who received their second vaccination before June 1, 2021, (i.e., before the end of the third wave of the pandemic in Bavaria) were significantly protected against COVID-19, both in multivariate analysis (*P*_r_ < 0.0001, Table 1), and compared to individuals who were vaccinated later (5.9%, 95% CI 5.1–6.8 vs. 9.0%, 95% CI 7.2–11.1, *P* = 0.002).

### Lifestyle-dependent COVID-19 risk and protective factors

**Fig. 4:**
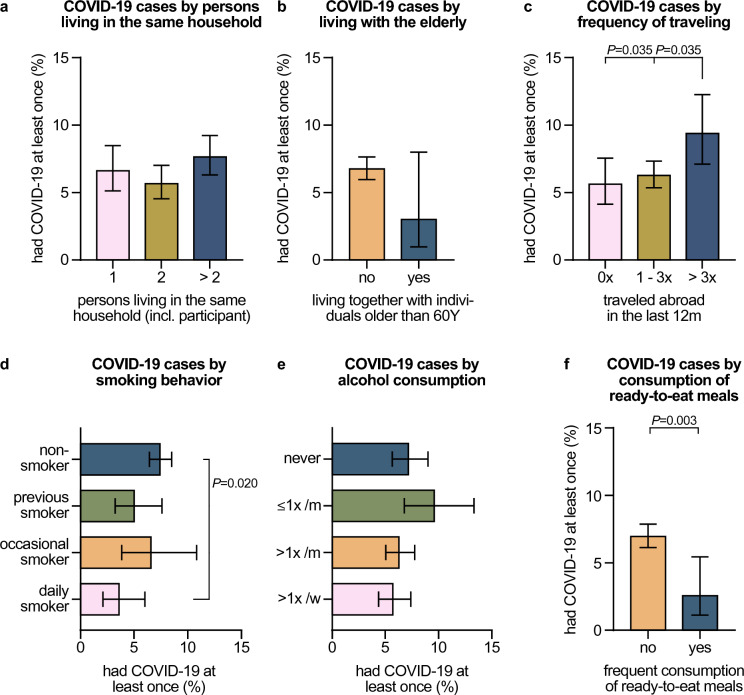
Lifestyle-dependent COVID-19 risk and protective factors. Percentages of all participants who had COVID-19 at least once (defined as being positive for nucleocapsid-specific anti-SARS-CoV-2 antibodies and/or reporting of ever having been tested positive for an acute SARS-CoV-2 infection by PCR) by **A** persons living in the same household as the respective study participant (including the participant), **B** elderly individuals (older than 60 years) living in the same household as the respective study participant, **C** traveling abroad in the last 12 month before sample collection, **D** smoking behavior, **E** alcohol consumption, **F** fast food consumption. Error bars indicate 95% confidence intervals obtained from Wilson corrected binominal testing. *P* values next to brackets indicate statistically significant differences between groups calculated using Fisher’s exact test (**B**, **F**) or Fisher’s exact test with Holm’s multiple testing correction (**A**, **C**–**E**). If no *P* values are given, no statistically significant differences were detected. *incl.* including

Regarding the living situation of participants, we found that the rate of individuals who have had COVID-19 was not influenced by the number of persons living in the same household as the participant (Fig. 4A). However, HCWs living with an elderly person aged 60 years or above showed a tendency towards lower rates of COVID-19 (3.0%, 95% CI 1.0–8.0) compared to others (6.8%, 95% CI 5.6–7.3, Fig. 4B). In line with this finding, living with the elderly was a statistically significant protective factor against COVID-19 in our multivariate data analysis (*P*_r_ = 0.0475, Table 1). Living with children and adolescents, on the contrary, did not influence the COVID-19 risk (Supplementary Table 1). The rates of individuals who have had COVID-19 were similar in participants reporting that they regularly engage in at least moderate physical activities compared to others (Supplementary Table 1).

Participants who reported traveling abroad more than three times within 1 year before inclusion had significantly higher COVID-19 rates (9.4%, 95% CI 7.1–12.3), both in multivariate analysis (*P*_r_ = 0.0088, Table 1), and compared to those who traveled less often (6.3%, 95% CI 5.4–7.3, *P* = 0.035), or not at all (5.6%, 95% CI 4.1–7.6, *P* = 0.035, Fig. 4C).

Smoking behavior influenced the COVID-19 risk in our study cohort. Individuals who indicated to smoke on a daily basis (i.e., frequent smokers) had significantly lower COVID-19 rates (3.6%, 95% CI 2.1–6.0) compared to non-smokers (7.4%, 95% CI 6.4–8.5, *P* = 0.020, Fig. 4D). Frequent smoking was, furthermore, a factor significantly associated with lower COVID-19 risk in our multivariate data analysis (*P*_r_ = 0.0088, Table 1). Participants who reported drinking alcohol more than once a week showed a tendency towards lower COVID-19 rates (5.7%, 95% CI 4.4–7.4) compared to others (Fig. 4E).

Finally, we wondered whether the diet of participants modulated their COVID-19 risk. Eating meat or fish on a regular basis, being pescatarian, vegetarian or vegan, avoiding dairy products or food containing lactose, gluten, peanuts and other nuts, or fish and shellfish had no impact on the COVID-19 risk in the study cohort (Supplementary Table 1). Similarly, taking vitamins, mineral supplements, or fish oil did not alter the infection rate (Supplementary Table 1). To our surprise, however, COVID-19 rates were significantly reduced in HCWs reporting regular consumption of ready-to-eat meals and processed foods, both in multivariate analysis (*P*_r_ = 0.0045, Table 1), and compared to individuals reporting no regular consumption of ready-to-eat meals (2.6%, 95% CI 1.1–5.4 vs. 7.0%, 95% CI 6.1–7.8, *P* = 0.003, Fig. 4F). In-depth analysis of participants indicating that they frequently eat read-to-eat meals and processed foods revealed that they are significantly younger than individuals who do not report this dietary habit (median 33 years vs. 39 years, *P* < 0.001), have a higher body mass index (median 23.9 vs. 23.2, *P* = 0.006), are more often of male gender (42% vs. 24%, *P* < 0.001), work more often in full-time employment (72% vs. 62%, *P* < 0.001), consume meat more frequently (36% vs. 13%, *P* < 0.01), live more often alone (40% vs. 23%, *P* < 0.01), and encompass a higher rate of daily smokers (17% vs. 11%, *P* < 0.01).

### Strength of association between potential modulators and COVID-19

**Fig. 5:**
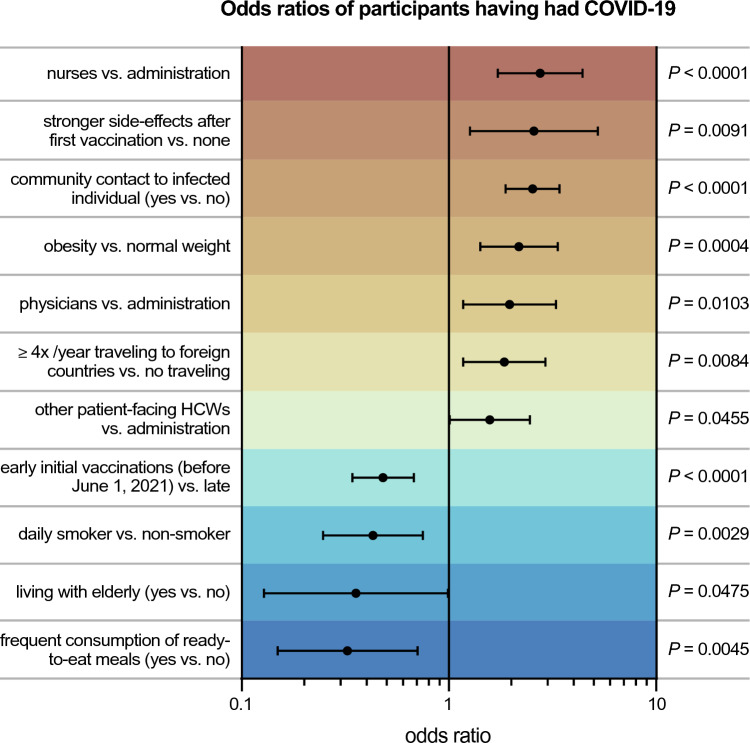
Strength of association between potential modulators and COVID-19. Depicted are odds ratios for the association between different factors and having had COVID-19 upon inclusion into the study calculated from multivariate logistic regression with backward elimination adjusted for gender and age. Error bars indicate 95% Wald confidence intervals. *HCWs* healthcare workers

In our study, we discovered potential risk and protective factors for COVID-19 that were statistically significant in multivariate analysis (Table 1). In order to compare the strength of the association between these factors and COVID-19, we calculated the odds of having had COVID-19, depending on reporting any of these risk and protective factors. Indicating being a nurse compared to working in administration had the highest age- and gender-adjusted odds ratio (OR) in our multivariate data analysis (OR 2.75, 95% CI 1.72–4.42, *P* < 0.0001, Fig. 5). Other factors with strong association to sub-acute or resolved COVID-19 were experiencing more intense adverse effects after the first vaccination (OR 2.57, 95% CI 1.27–5.23, *P* = 0.0091), reporting of contacts to infected individuals in the community (OR 2.53, 95% CI 1.88–3.42, *P* < 0.0001), and being obese compared to having a normal body weight (OR 2.18, 95% CI 1.42–3.35, *P* = 0.0004, Fig. 5).

Reporting frequent consumption of ready-to-eat meals showed the strongest negative association to having experienced COVID-19 (OR 0.32, 95% CI 0.15–0.71, *P* = 0.0045, Fig. 5). Similarly, living with elderly persons (OR 0.36, 95% CI 0.13–0.99, *P* = 0.0475), and daily smoking behavior compared to being a non-smoker (OR 0.43, 95% CI 0.25–0.75, *P* = 0.0029) shared a strong negative association to COVID-19 (Fig. 5).

## Discussion

In a cross-sectional study, we identified several factors that modulate the COVID-19 risk in HCWs. Among others, working in an occupation with direct contact to patients and exposure to SARS-CoV-2-infected colleagues were occupational COVID-19 risk factors. Individuals had a significantly lower risk of infection if they had completed their first two COVID-19 vaccinations prior June 2021. On the contrary, experiencing more severe adverse effects after the first vaccination and being obese were associated with higher COVID-19 rates. Moreover, the lifestyle of participants impacted their risk to be infected with SARS-CoV-2: individuals who oftentimes traveled abroad during the pandemic showed higher COVID-19 rates and, conversely, participants living together with elderly persons, frequent smokers, and those who regularly consumed ready-to-eat meals and processed foods had lower infection rates. To our knowledge, this study for the first time provides evidence that traveling abroad, residing with the elderly and frequent ingestion of ready-to-eat meal are associated with altered COVID-19 risk.

Approximately, one-third of the participants who have had COVID-19 upon inclusion into the study never had been tested positive for an acute SARS-CoV-2 infection. We hypothesize that there were many asymptomatic cases among these convalescent individuals. Indeed, similar rates of about 30% of asymptomatic COVID-19 were described in HCWs after the primary wave of the COVID-19 pandemic [11].

Our finding of increased COVID-19 risk in patient care healthcare professionals, especially in nurses, has been described in other studies evaluating risk factors for SARS-CoV-2 infection [11, 12], and BI in HCWs [13–15]. On a similar note, the risk factor of exposure to infected individuals, which we found in this study, was also reported by others [11, 16, 17]. Regarding occupation-associated exposure to SARS-CoV-2, we found that contacts to infected colleagues, in particular, elevated the COVID-19 risk in HCWs. Conceivably, viral transmission between co-workers occurred during breaks when healthcare professionals spent time together without wearing the appropriate personal protective equipment. The strongest factor associated with high rates of participants having had COVID-19 in our cohort, however, was non-occupational exposure to infected individuals in the community, in line with results from other studies [11, 18, 19].

Three of the factors that we discovered to be associated with increased COVID-19 rates likely were no bona fide risk factors, but rather indicators for prior SARS-CoV-2 infection: individuals who were suspicious of having COVID-19 (e.g., because they experienced symptoms) might have sought PCR testing at the hospital more often. This potentially explains the higher COVID-19 rates in HCWs who were tested more than four times in the previous four weeks compared to individuals who were tested less frequently. Similarly, “reverse causality” is a possible reason for increased COVID-19 rates in participants who reported more intense adverse effects after the first vaccination. We hypothesize that individuals who had COVID-19 before the first vaccination showed a stronger reaction to the exposure with the spike antigen compared to naïve individuals, in line with results from recent studies [20, 21]. Finally, we observed elevated SARS-CoV-2 infection rates among double-vaccinated HCWs who were reluctant to receive a third dose of the COVID-19 vaccine. Until November 29, 2021, i.e., during the first phase of the study’s recruitment period, there were no official recommendations in Germany for convalescent persons who were vaccinated twice to receive a third dose of the vaccine. This may have contributed to the finding that individuals who knew that they have had COVID-19 were more reluctant towards the third vaccination.

Our finding that individuals who received their initial two COVID-19 vaccinations before June 1, 2021, had lower COVID-19 rates compared to others, indicates a protective effect of earlier immunization. It is likely that such early vaccinated HCWs were better protected against SARS-CoV-2 infection in spring 2021 during the third pandemic wave in Germany, when SARS-CoV-2 VoC alpha caused a considerable increase of COVID-19 cases in Bavaria, compared to other participants who were not yet vaccinated twice at that time. From the end of the third pandemic wave until the rise of the fourth (which was coincidental with the period of sample collection for our study, Fig. 1A) COVID-19 incidences and the risk for infection were comparably low and, thus, vaccination-associated protection potentially impacted the overall rates of individuals who have had COVID-19 at least once to a lesser degree. In conclusion, this finding highlights the potential benefits of rapid implementation of vaccination programs to counteract the COVID-19 pandemic which could, in turn, help refining strategies to combat emerging infectious diseases in future.

Analyzing the influence of health conditions reported by the participants, we found obesity to increase the COVID-19 risk. Obesity often has been associated with increased susceptibility to SARS-CoV-2 infection [22–25], and BI [26, 27], as well as severe COVID-19 [28–30]. In addition, we discovered a trend towards reduced COVID-19 rates in underweight individuals, which to our knowledge had not been described before. Larger and more focused studies could be useful to investigate the role of low body weight on the susceptibility to this and potentially also other viruses.

For the evaluation of lifestyle-dependent modulators of the COVID-19 risk, we first prompted the participants to report their living situation. We observed that living with elderly persons was significantly associated to low SARS-CoV-2 infection rates. Old age is a widely accepted risk factor for severe COVID-19, and COVID-19-associated death [31–34]. Thus, it is likely that participants who lived together in the same household with an elderly individual were more careful and applied containment measures such as social distancing more strictly compared to others, ultimately leading to decreased COVID-19 rates in this group.

Smoking behavior was another lifestyle that we discovered to be associated with decreased COVID-19 risk, conforming the findings of others [11, 35, 36].

Frequent traveling to foreign countries during the pandemic was associated with increased SARS-CoV-2 infection rates in our study cohort. To our knowledge, we are the first to show evidence on traveling potentially being a COVID-19 risk factor. Conceivably, traveling abroad, especially by airplane, is concomitant with an increased number of close contacts to other individuals thereby likely increasing the chances of SARS-CoV-2 transmission [37]. Moreover, individuals traveling frequently during this phase of the pandemic despite travel restrictions and recommendations to limit international traveling might generally be less cautious and, thus, more likely to contract this airborne infection.

Frequent consumption of ready-to-eat meals and processed foods was most strongly associated with low COVID-19 rates in our multivariate data analysis. Consumption of such foods, thus far, was never described as a potential protective factor against COVID-19 but, on the contrary, it was observed that higher intake of ultra-processed food potentially increases the COVID-19 risk [38]. In our sub-analysis, participants reporting this eating pattern displayed specific characteristics related to biological factors, medicinal conditions, the work and living situation, as well as other dietary habits and smoking behavior. The findings in this study show an association between dietary habits and SARS-CoV-2 infection rates, but no causality can be derived from our observations. Future studies should, thus, characterize whether there are biological mechanisms, e.g., mediated by defined food-associated metabolites, or other factors that can explain the influence of dietary habits on COVID-19 risk.

Our study has several limitations. First, the cross-sectional approach and data collection did not allow for differentiation between participants who have had a SARS-CoV-2 infection prior to vaccination and those with a BI with absolute certainty. Furthermore, we were unable to elucidate whether participants had COVID-19 only once or several times. The risk factors for infection, however, might differ in non-vaccinated, naïve individuals compared to convalescent and vaccinated ones. Another limitation is that the study was performed before the emergence of SARS-CoV-2 VoC omicron. Omicron and its sub-variants show considerable evolution and comparably strong immune escape, thereby, potentially also altering the risk factors for infection. This study relies not only on diagnostical results but also on self-reported data, which is potentially biased. The observations made in this study show the association between different occupational, as well as non-occupational factors in HCWs and altered COVID-19 rates. These associations might, in part, be influenced by confounding variables that were not included in the study questionnaire or, because of other reasons, could not be explored in greater detail in this study. Finally, the HCW cohort evaluated in this study, even though it is quite divers, does not fully reflect the general population. There is, for example, a higher percentage of women among the study participants and more highly educated individuals compared to the general population of Germany [39]. Moreover, the cohort’s age distribution does not fully match the age demographic in the state of Bavaria, Germany [39]. Thus, some of the non-occupational modulators of the COVID-19 risk discovered here might not be applicable to the general population. However, obesity and smoking behavior, which we found as modulators of the susceptibility to SARS-CoV-2 infection in our participants, are well-established non-occupational factors influencing the COVID-19 risk [22–25, 35, 36]. We, therefore, anticipate that other lifestyle-dependent factors associated with altered COVID-19 risk that we newly discovered in this study are also relevant for the general public. The HCWs in this cohort largely received the same COVID-19 mRNA vaccine in a thoroughly organized and synchronized fashion and, furthermore, were highly motivated to participate in the study and contribute to the research presented here, so much that the majority of them answered the highly detailed questionnaire. Evidence from other studies show that such a high degree of adherence is difficult to achieve in participants recruited from the general population [41].

Our study underlines the importance of certain, previously described occupational COVID-19 risk factors in HCWs. Moreover, we show that the susceptibility to SARS-CoV-2 infection can be shaped by preexisting medical conditions and the individual lifestyle, including the living situation, traveling and smoking behavior as well as dietary habits. Besides being a venture point for more in-depth biological and behavioral studies, our findings may guide the installation and refinement of containment measures to improve the protection of HCWs, patients and visitors in hospitals and, furthermore, aid the identification of additional risk groups and vulnerable individuals in the general population.

### Supplementary Information

Below is the link to the electronic supplementary material.

Supplementary file1 (PDF 876 KB)

## Data Availability

The data used and/or analyzed during the current study are available from the corresponding author on reasonable request.
