# Supplementary material for: Dietary habits, traveling and the living situation potentially influence the susceptibility to SARS-CoV-2 infection: results from healthcare workers participating in the RisCoin Study
Source: Infection. 2024 Mar 4;52(4):1425–37. doi: 10.1007/s15010-024-02201-4 (PMC11289231; doi:10.1007/s15010-024-02201-4)
Supplement: Supplementary file 1 — Supplementary file1 (PDF 876 KB) [file 15010_2024_2201_MOESM1_ESM.pdf]

Supplementary Information:

Dietary habits, travelling and the living situation potentially influence the susceptibility to SARS-CoV-2 infection: results from healthcare workers participating in the RisCoin Study

Paul R. Wratil, Thu Giang Le Thi, Andreas Osterman, Irina Badell, Melanie Huber, Ana Zhelyazkova, Sven P Wichert, Anna Litwin, Stefan Hörmansdorfer, Frances Strobl, Veit Grote, Tarek Jebrini, Helga P Török, Veit Hornung, Alexander Choukér, Berthold Koletzko, Kristina Adorjan, Sibylle Koletzko, Oliver T. Keppler on behalf of the RisCoin study group

Corresponding Authors:

Kristina Adorjan (kristina.adorjan@med.uni-muenchen.de)

Sibylle Koletzko (sibylle.koletzko@med.uni-muenchen.de)

Oliver T. Keppler (keppler@mvp.lmu.de)

Supplementary Table 1 (part 1). Answers from the study questionnaire, percentages of participants that gave a certain answer having had COVID-19 (defined as being positive for nucleocapsid-specific anti-SARS-CoV-2 antibodies and/or reporting of ever having been tested positive for an acute SARS-CoV-2 infection by PCR), and pairwise statistical evaluation

Item	Answers	Total	% had COVID-19	95% CI	Pairwise comparisons	*P*-value
Gender	Male	946	7.1	5.6 – 9.0	n.s.	
	Female	2,741	6.5	5.6 – 7.5		
Age group	18 – 30 years	1,068	7.9	6.4 – 9.7	n.s.	
	31 – 40 years	930	5.9	4.5 – 7.7		
	41 – 50 years	672	8.0	6.1 – 10.4		
	51 – 60 years	756	5.0	3.6 – 6.9		
	> 60 years	270	5.2	3.0 – 8.7		
Weight class calculated from the self-reported body mass index	Underweight	106	3.8	1.2 – 9.9	n.s.	
	Normal weight	2,329	6.4	5.5 – 7.5		
	Pre-obesity	887	6.1	4.6 – 7.9		
	Obesity	374	9.9	7.1 – 13.5		
Formal education	Middle school diploma	362	6.4	4.2 – 9.5	n.s.	
	High school diploma	884	7.4	5.8 – 9.3		
	Completed apprenticeship	737	6.8	5.1 – 8.9		
	University degree	1,666	6.1	5.0 – 7.4		
Occupation	Nurse	868	8.4	6.7 – 10.5	Nurses vs. administration	0.024
	Physician	664	7.2	5.4 – 9.5		
	Administration	721	4.6	3.2 – 6.4		
	Other patient care occupation	721	6.4	5.1 – 8.1		
	Non-patient care occupation	1,133	5.1	3.0 – 8.5		
Regularly experiencing patient contacts	No	1,384	5.5	4.4 – 6.9	No vs. yes	0.0341
	Yes	2,310	7.3	6.3 – 8.5		
Workplace	Wards w/ COVID-19 patients	291	8.6	5.7 – 12.6	n.s.	
	Wards w/o COVID19 patients	859	7.6	5.9 – 9.6		
	Emergency units	76	9.2	4.1 – 18.6		
	Outpatient clinics	442	8.1	5.8 – 11.2		
	Other clinical departments	642	5.6	4.0 – 7.8		
	Non-clinical departments	1,384	5.5	4.4 – 6.9		
Contact to SARS-CoV-2-infected individuals	No	1,962	4.9	4.0 – 6.0	No vs. yes	< 0.0001
	Yes	1,715	8.6	7.3 – 10.0		

Binominal 95% confidence intervals (95% CI) were calculated using the Wilson score interval. Differences between groups were tested for their statistical significance using Fisher's exact test, in case of multiple variables with Holm's testing corrections. n.s. – no statistical significance in pairwise comparisons

Supplementary Table 1 (part 2). Answers from the study questionnaire, percentages of participants that gave a certain answer having had COVID-19 (defined as being positive for nucleocapsid-specific anti-SARS-CoV-2 antibodies and/or reporting of ever having been tested positive for an acute SARS-CoV-2 infection by PCR), and pairwise statistical evaluation

Item	Answers	Total	% had COVID-19	95% CI	Pairwise comparisons	*P*-value
Contact to infected colleague	No	2,849	6.0	5.2 – 7.0	No vs. yes	0.0110
	Yes	828	8.6	6.8 – 10.7		
Contact to infected patient at work	No	2,770	6.3	5.4 – 7.3	n.s.	
	Yes	907	7.6	6.0 – 9.6		
Contact to infected individual in the community	No	3,077	5.4	4.6 – 6.3	No vs. yes	< 0.0001
	Yes	600	12.8	10.3 – 15.8		
SARS-CoV-2 test performed in the last 4w before inclusion	None	1,493	6.2	5.0 – 7.5	1 – 4 vs. > 4	0.057
	1 – 4	1,949	6.5	5.5 – 7.7		
	> 4	254	10.2	6.9 – 14.8		
Adverse effects after the 1st vaccination	None	2,361	6.3	5.3 – 7.3	n.s.	
	Mild	1,246	7.0	5.7 – 8.6		
	More severe	89	11.2	5.8 – 20.1		
Adverse effects after the 2nd vaccination	None	1,582	6.6	5.5 – 8.0	n.s.	
	Mild	1,752	7.0	5.9 – 8.3		
	More severe	359	4.7	2.9 – 7.6		
Adverse effects after the 3rd vaccination (partially reported after inclusion)	None	600	6.2	4.4 – 8.5	n.s.	
	Mild	693	5.3	3.8 – 7.4		
	More severe	396	4.0	2.4 – 6.6		
Had received the 3rd vaccination at inclusion	Yes	282	5.0	2.8 – 8.4	n.s.	
	No	3,414	6.8	6.0 – 7.7		
Acceptance for the 3rd vaccination	Yes	3,356	6.4	5.6 – 7.3	No vs. yes	0.0394
	No	340	8.8	6.1 – 12.5		
Vaccinated against influenza	During the last season	1,932	6.3	5.2 – 7.5	n.s.	
	Not during the last season	660	7.3	5.5 – 9.6		
	Never	1,023	6.8	5.4 – 8.6		
Has cardiovascular disease	No	3,422	6.7	5.9 – 7.6	n.s.	
	Yes	273	5.5	3.2 – 9.1		
Has chronic pulmonary disease	No	3,482	6.6	5.8 – 7.5	n.s.	
	Yes	213	7.0	4.1 – 11.6		
Has diabetes mellitus	No	3,626	6.5	5.8 – 7.4	n.s.	
	Yes	69	11.6	5.5 – 22.1		

Binominal 95% confidence intervals (95% CI) were calculated using the Wilson score interval. Differences between groups were tested for their statistical significance using Fisher's exact test, in case of multiple variables with Holm's testing corrections. n.s. – no statistical significance in pairwise comparisons

Supplementary Table 1 (part 3). Answers from the study questionnaire, percentages of participants that gave a certain answer having had COVID-19 (defined as being positive for nucleocapsid-specific anti-SARS-CoV-2 antibodies and/or reporting of ever having been tested positive for an acute SARS-CoV-2 infection by PCR), and pairwise statistical evaluation

Item	Answers	Total	% had COVID-19	95% CI	Pairwise comparisons	*P*-value
Has metabolic disorder	No	3,592	6.6	5.8 – 7.5	n.s.	
	Yes	103	7.8	3.7 – 15.2		
Has thyroid dysfunction	No	3,107	6.7	5.9 – 7.7	n.s.	
	Yes	586	6.1	4.4 – 8.5		
Has chronic renal disease	No	3,674	6.6	5.9 – 7.5	n.s.	
	Yes	19	5.3	0.3 – 28.1		
Has hepatic or gastrointestinal disease	No	3,612	6.8	6.0 – 7.6	n.s.	
	Yes	80	1.3	0.1 – 7.7		
Has chronic neurological disorder	No	3,612	6.5	5.8 – 7.4	n.s.	
	Yes	80	11.3	5.6 – 20.8		
Has Cancer	No	3,569	6.7	5.9 – 7.6	n.s.	
	Yes	123	4.1	1.5 – 9.7		
Has chronic disease of the immune system	No	3,653	6.7	5.9 – 7.5	n.s.	
	Yes	39	2.6	0.1 – 15.1		
Has any allergies	No	2,115	7.2	6.2 – 8.4	n.s.	
	Yes	1,577	5.8	4.7 – 7.1		
Has allergic rhinoconjunctivitis (caused by pollen or house dust)	No	2,573	6.8	5.9 – 7.9	n.s.	
	Yes	1,119	6.2	4.9 – 7.8		
Has drug allergies	No	3,263	6.7	5.9 – 7.6	n.s.	
	Yes	429	5.8	3.9 – 8.6		
Has food allergies	No	3,550	6.7	5.9 – 7.6	n.s.	
	Yes	142	3.5	1.3 – 8.4		
Has allergies against bee or wasp poison	No	3,600	6.7	5.9 – 7.5	n.s.	
	Yes	92	4.3	1.4 – 11.4		
Has contact allergies against chemical compounds	No	3,463	6.7	5.9 – 7.6	n.s.	
	Yes	229	5.2	2.9 – 9.2		
Has ever experienced an allergic shock	No	3,547	6.7	5.9 – 7.5	n.s.	
	Yes	145	5.5	2.6 – 10.9		
Takes immunosuppressive medication	No	3,629	6.6	5.8 – 7.4	n.s.	
	Yes	66	10.6	4.7 – 21.2		
Regular intake of vitamin supplements	No	3,357	6.6	5.8 – 7.5	n.s.	
	Yes	287	5.6	3.3 – 9.1		

Binominal 95% confidence intervals (95% CI) were calculated using the Wilson score interval. Differences between groups were tested for their statistical significance using Fisher's exact test, in case of multiple variables with Holm's testing corrections. n.s. – no statistical significance in pairwise comparisons

Supplementary Table 1 (part 4). Answers from the study questionnaire, percentages of participants that gave a certain answer having had COVID-19 (defined as being positive for nucleocapsid-specific anti-SARS-CoV-2 antibodies and/or reporting of ever having been tested positive for an acute SARS-CoV-2 infection by PCR), and pairwise statistical evaluation

Item	Answers	Total	% had COVID-19	95% CI	Pairwise comparisons	*P*-value
Regular intake of fish oil	No	3,484	6.5	5.7 – 7.3	n.s.	
	Yes	160	8.1	4.6 – 13.8		
Regular intake of mineral supplements	No	3,289	6.3	5.5 – 7.2	n.s.	
	Yes	355	8.5	5.9 – 12.0		
Regular intake of vegetables and fruits	No	1,531	6.1	5.0 – 7.4	n.s.	
	Yes	2,165	7.0	6.0 – 8.2		
Daily or almost daily consumption of meat	No	3,144	6.7	5.9 – 7.7	n.s.	
	Yes	552	6.0	4.2 – 8.4		
Frequent consumption of ready-to-eat meals	No	3,423	7.0	6.1 – 7.9	No vs. yes	0.0033
	Yes	273	2.6	1.1 – 5.4		
Pescetarian diet	No	3,324	6.6	5.8 – 7.5	n.s.	
	Yes	372	7.3	4.9 – 10.5		
Frequent consumption of fish	No	3,136	6.8	5.9 – 7.7	n.s.	
	Yes	560	5.9	4.2 – 8.3		
Vegan diet	No	3,580	6.7	5.9 – 7.6	n.s.	
	Yes	116	5.2	2.1 – 11.4		
Weight control diet	No	3,545	6.6	5.8 – 7.5	n.s.	
	Yes	151	6.6	3.4 – 12.2		
Avoids particular foods due to allergies/intolerances	No	3,461	6.8	6.0 – 7.7	n.s.	
	Yes	235	4.7	2.5 – 8.5		
Individuals living in the same household as the participant (incl. the participant)	1	907	6.6	5.1 – 8.5	n.s.	
	2	1,431	5.7	4.5 – 7.0		
	> 2	1,335	7.6	6.3 – 9.2		
Living with children	No	2,681	6.4	5.5 – 7.4	n.s.	
	Yes	992	7.2	5.7 – 9.0		
Living with adolescents aged 14 – 18	No	3,348	6.4	5.6 – 7.3	n.s.	
	Yes	325	8.9	6.2 – 12.7		
Living with elderly aged 60 and above	No	3,540	6.8	6.0 – 7.6	n.s.	
	Yes	133	3.0	1.0 – 8.0		
Living in a community facility or residential home	No	3,429	6.4	5.6 – 7.2	n.s.	
	Yes	261	9.6	6.4 – 14.0		

Binominal 95% confidence intervals (95% CI) were calculated using the Wilson score interval. Differences between groups were tested for their statistical significance using Fisher's exact test, in case of multiple variables with Holm's testing corrections. n.s. – no statistical significance in pairwise comparisons

Supplementary Table 1 (part 5). Answers from the study questionnaire, percentages of participants that gave a certain answer having had COVID-19 (defined as being positive for nucleocapsid-specific anti-SARS-CoV-2 antibodies and/or reporting of ever having been tested positive for an acute SARS-CoV-2 infection by PCR), and pairwise statistical evaluation

Item	Answers	Total	% had COVID-19	95% CI	Pairwise comparisons	*P*-value
Travelled abroad in the last 12 months before inclusion	Never	766	5.6	4.1 – 7.6	Never vs. > 3x	0.0035
	1 – 3x	2,391	6.3	5.4 – 7.3	1 – 3x vs. > 3x	0.0035
	> 3x	533	9.4	7.1 – 12.3		
Has at least moderate physical activity	No	673	5.8	4.2 – 7.9	n.s.	
	1x per week	1,168	7.9	6.4 – 9.6		
	> 1x per week	1,849	6.1	5.0 – 7.3		
Smoking behavior	Non-smoker	2,565	7.4	6.4 – 8.5	Non-smoker vs. daily smoker	0.02
	Previous smoker	439	5.0	3.2 – 7.6		
	Occasional smoker	228	6.6	3.9 – 10.8		
	Daily smoker	416	3.6	2.1 – 6.0		
Alcohol consumption	Never	977	7.2	5.7 – 9.0	n.s.	
	< 1x per month	344	9.6	6.8 – 13.3		
	> 1x per month	1,306	6.3	5.1 – 7.8		
	> 1x per week	981	5.7	4.4 – 7.4		
Alcohol consumption	At least occasionally	2,655	6.4	5.6 – 7.5	n.s.	
	Previous consumption	92	4.3	1.4 – 11.4		
	No consumption	943	7.2	5.7 – 9.1		
Full-time employment	No	1,372	6.9	5.6 – 8.4	n.s.	
	Yes	2,320	6.5	5.6 – 7.6		
Double-vaccinated before June 1, 2021	No	858	9.0	7.2 – 11.1	No vs. yes	0.0022
	Yes	2,838	5.9	5.1 – 6.8		

Binominal 95% confidence intervals (95% CI) were calculated using the Wilson score interval. Differences between groups were tested for their statistical significance using Fisher's exact test, in case of multiple variables with Holm's testing corrections. n.s. – no statistical significance in pairwise comparisons
